# Age-Related Hearing Loss: Sensory and Neural Etiology and Their Interdependence

**DOI:** 10.3389/fnagi.2022.814528

**Published:** 2022-02-17

**Authors:** Karen L. Elliott, Bernd Fritzsch, Ebenezer N. Yamoah, Azel Zine

**Affiliations:** ^1^Department of Biology, University of Iowa, Iowa City, IA, United States; ^2^Department of Physiology and Cell Biology, School of Medicine, University of Nevada, Reno, NV, United States; ^3^LBN, Laboratory of Bioengineering and Nanoscience, University of Montpellier, Montpellier, France

**Keywords:** OHC, IHC, SGN, cochlear nuclei, SOC

## Abstract

Age-related hearing loss (ARHL) is a common, increasing problem for older adults, affecting about 1 billion people by 2050. We aim to correlate the different reductions of hearing from cochlear hair cells (HCs), spiral ganglion neurons (SGNs), cochlear nuclei (CN), and superior olivary complex (SOC) with the analysis of various reasons for each one on the sensory deficit profiles. Outer HCs show a progressive loss in a basal-to-apical gradient, and inner HCs show a loss in a apex-to-base progression that results in ARHL at high frequencies after 70 years of age. In early neonates, SGNs innervation of cochlear HCs is maintained. Loss of SGNs results in a considerable decrease (~50% or more) of cochlear nuclei in neonates, though the loss is milder in older mice and humans. The dorsal cochlear nuclei (fusiform neurons) project directly to the inferior colliculi while most anterior cochlear nuclei reach the SOC. Reducing the number of neurons in the medial nucleus of the trapezoid body (MNTB) affects the interactions with the lateral superior olive to fine-tune ipsi- and contralateral projections that may remain normal in mice, possibly humans. The inferior colliculi receive direct cochlear fibers and second-order fibers from the superior olivary complex. Loss of the second-order fibers leads to hearing loss in mice and humans. Although ARHL may arise from many complex causes, HC degeneration remains the more significant problem of hearing restoration that would replace the cochlear implant. The review presents recent findings of older humans and mice with hearing loss.

## Introduction

Age-related hearing loss (ARHL) is a significant issue that leads to a reduced hearing perception in older humans. Half of the adult men over the age of 70 have ARHL (Roccio et al., 2020; Yamoah et al., 2020; Wu et al., 2021), which makes it a significant public health concern. The World Health Organization (WHO) predicts that there will be approximately 1 billion people over the age of 65 by the year 2050. Thus, ARHL is poised to be a substantial problem. ARHL involves a combination of conductive hearing loss (middle ear defects) and/or neurosensory hearing loss (loss of hair cells or neurons). It may also involve second-order neuron functional decline in the cochlear nuclei (CN) and associated superior olivary complex (SOC) (Makary et al., 2011; Lang, 2016; Caspary and Llano, 2018; Fattal et al., 2018; Dubno, 2019; Syka, 2020). Roughly 30% of women and men older than 65 have a reduced hearing sensitivity (Homans et al., 2017; Eggermont, 2019) affecting approximately 600 million people worldwide who may develop a progressive neurosensory hearing loss. Overall, one can imply that about 90% of people aged 90 years and older will eventually suffer from overt hearing loss unilaterally, affecting at least one ear (Sheffield and Smith, 2019). Aging also decreases sound localization accuracy and the ability to detect weak signals in noise.

Additionally, the repeated noise exposure begets noise-induced hearing loss (NIHL), which may exacerbate ARHL. These deficits may also indirectly affect cognition through decreased social activities and communication (Dubno, 2019; Eckert et al., 2019; Syka, 2020). A better understanding of the aging auditory system mechanisms and eventually replacing lost hair cells and sensory neurons to maintain hearing is required to help those with hearing replacement, including cochlear implants.

**Figure 1:**
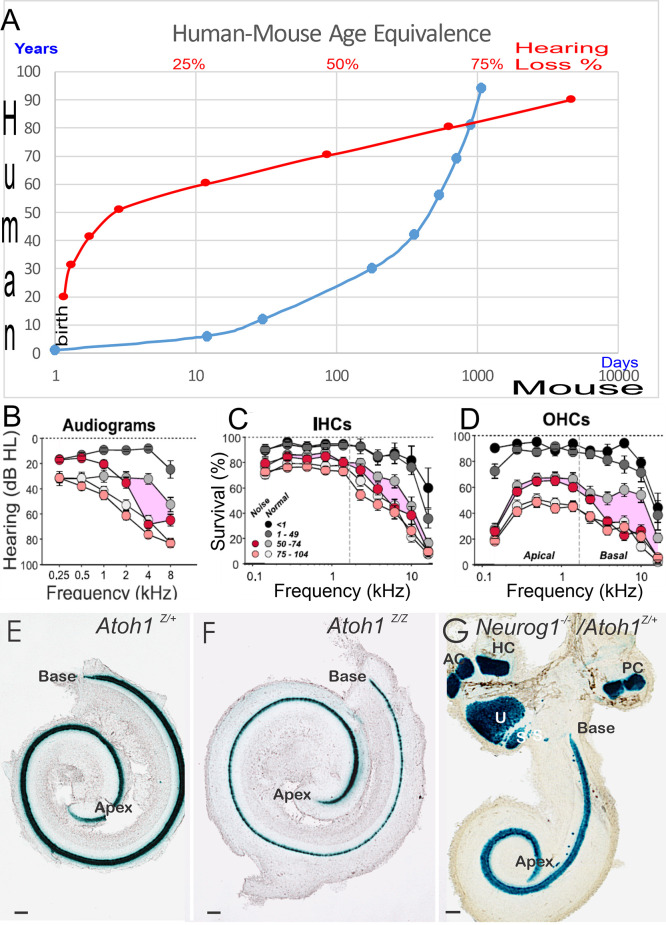
Panel **(A)** provides an overview of a 70-year-old human comparable to a 2-year-old mouse. The age group in humans displays profound hearing decay, shown as percent (%) of hearing loss (**A**, in red) follow-on hearing loss of 90-year-old humans. Limited restoration is attempted that has focused on 2-year or mostly younger mice, that is driven by the cost of keeping animals to old age and is providing a lack of decent models. Audiograms **(B)** and histology **(C,D)** demonstrated the loss of IHC **(C)**, OHC **(D)**, and the progressive threshold reduction in older people shown with the audiogram **(B)**. Note that people exposed to noise have an additional loss in hair cells **(C,D)** and show a threshold reduction in the audiogram **(B)** in the middle age of 50–74 years old. In newborn mice, we demonstrate the phenotype of *Atoh1* LacZ in control mice **(E)** Atoh1LacZ/LacZ null mice **(F)** is comparable (i.e., a single row of undifferentiated IHCs). Compared to *Neurog1* null mice, a shorter cochlea has outliers in the GER area **(G)**. Data were taken from the Jackson lab, WHF, and Fritzsch et al. (2005); Matei et al. (2005); Sheffield and Smith (2019); and Wu et al. (2021). Scale bars: 100 μm **(E–G)**.

Currently, mouse models are the most common model of ARHL. The model appears to have limited transformation of supporting cells into HCs (Walters et al., 2017; Yamashita et al., 2018; Roccio et al., 2020; Li et al., 2020). In contrast, age-related hearing loss is understudied compared to earliest hair cell replacements (Zine et al., 2014, 2021; Devare et al., 2018; Yamoah et al., 2020). Notably, we realize that the stages of mouse development are different compared to humans (Lim and Brichta, 2016; Wang et al., 2018): a 2-year-old mouse is equivalent to a ~70-year-old human (Figure 1A). The corresponding human age to which hair cells are lost, leading to a particularly profound hearing loss in a mouse (Rauch et al., 2001; Kusunoki et al., 2004; Eggermont, 2019; Sheffield and Smith, 2019) depends upon the specific mouse strain (Zheng et al., 1999). Age-related hearing loss and changes in hearing of aged people have been identified at many levels across the entire auditory system, from the cochlear hair cells to the CN to the SOC. Reviews have reported central auditory dysfunction, including the downregulation of synaptic inhibition due to peripheral deafferentation and maladaptive plasticity. Aging-related decreases in synaptic inhibition (Stebbings et al., 2016; Caspary and Llano, 2018) may be triggered by high metabolic demands that lead to inhibitory neurons that being affected, while the relative to the decline of their excitatory counterparts (Kann et al., 2014), yielding an inhibitory-excitatory imbalance. Metabolic demands may be higher in the auditory brainstem, including the SOC, due to having high firing rates of neurons (Sanes and Rubel, 1988; Kujawa and Liberman, 2009), potentially making them more vulnerable to aging-related declines. Recent data suggest that lateral superior olivary neurons (LSON) are more resistant to metabolic stress than SOC overall (Brosel et al., 2018).

Hair cell loss is one of the early signs leading to the progressive worsening of the organ of Corti (Pauley et al., 2008; Taylor et al., 2012; Kersigo and Fritzsch, 2015; Herranen et al., 2020; Wu et al., 2021). The loss of spiral ganglion neurons (SGN), cochlear nuclei (AVCN, PVCN, DCN), and superior olivary complex (SOC) may follow or may occur in tandem. A common consequence of hair-cell loss is a flat epithelium (Shibata et al., 2010), which constitutes a predominated clinical situation of hearing loss that requires a future cure (Yamasoba et al., 2013; Yang et al., 2015; Homans et al., 2017; Revuelta et al., 2017; Dawson and Bowl, 2019; Dubno, 2019; Lopez-Juarez et al., 2019). Several studies have focused on various aspects of age-related hearing loss prevention, while others work toward the therapy (Schilder et al., 2019; Eshraghi et al., 2020; Roccio et al., 2020; Sekiya and Holley, 2021). For instance, cochlear implants (CI) are effectively used to functionally replace hair cells (Gantz et al., 2018, 2005). In addition, current approaches are attempting to define in detail the genetic basis of ARHL (Lewis M. A. et al., 2018). Improved hearing loss measurement (Simpson et al., 2019; Cassarly et al., 2020) will guide the restoration of hair cells to ameliorate hearing loss.

The presented review will describe mouse mutants to endorse a genetic model with a flat auditory epithelium and associated loss of sensory neurons of the ear and the brainstem. We aim through this approach to inspire innovative research and novel interventions by framing the hallmarks of aging (Campisi, 2013; López-Otín et al., 2013; Sekiya and Holley, 2021) to improve the upcoming translatability of present restoration challenges. Developing those leads to novel mouse models to deal with the strengths and weaknesses. It would be helpful to develop restoration strategies that are temporally mountable from postnatal to old age. Age-dependent dysregulation of the cochlear duct endolymph composition is a problem (Dubno, 2019) that aggravates neuronal loss (Lang, 2016; Elliott et al., 2021b) and are other avenues of investigation. Their possible restoration for hearing perfection is being explored (Gantz et al., 2018; Yamoah et al., 2020). We focus on hair cells, sensory neurons, cochlear nuclei, and the MOC innervation as late-age-related restoration targets.

## Hair Cell Losses Begin in The Outer Hair Cells Followed by Inner Hair Cell Loss

Hair cells and supporting cells form the functional cellular assembly in the organ of Corti in the inner ear (Fritzsch et al., 2006; Groves and Fekete, 2017). In mice, hair-cells exit the cell cycle around embryonic days E12–14 in an apex to base progression (Ruben, 1967; Matei et al., 2005). In humans, this happens around gestational weeks GW12 (Locher et al., 2013). Many transcription factors and other genes are involved in cochlear hair cell development. For instance, *Eya1/Six1* early gene expression (Zou et al., 2004; Ahmed et al., 2012; Li et al., 2020; Xu et al., 2021) is followed by *Sox2* (Kiernan et al., 2005; Dvorakova et al., 2016, 2020). Other genes are needed such as *Gata3* (Karis et al., 2001; Duncan and Fritzsch, 2013; Walters et al., 2017), *Pax2* (Bouchard et al., 2010; Kempfle and Edge, 2014), *Shh* (Riccomagno et al., 2005; Muthu et al., 2019), and *miR-183* (Pierce et al., 2008; Kersigo et al., 2011). In addition, we know of several *Lim* domain factors such as *Isl1* (Huang et al., 2008; Chumak et al., 2016) and *Lhx3* (Hertzano et al., 2007) that are needed for normal development of the cochlear hair cells. A unique loss of *Lmx1a* (Huang et al., 2008, 2018; Nichols et al., 2008, 2020) and its interaction with *Lmx1b* (Chizhikov et al., 2021) regulates the development of all cochlear hair cells, comparable to deletions of either* Pax2* (Bouchard et al., 2010) or *Shh* (Muthu et al., 2019). Another subset of early transcription factors is known for proliferation, such as *p27^kip^* (Löwenheim et al., 1999; Zine et al., 2014), n-*Myc* (Kopecky et al., 2011), and *Prox1* (Fritzsch et al., 2010).

*Atoh1* expression is an essential early step of all inner ear hair cell differentiation (Bermingham et al., 1999; Fritzsch et al., 2005; Pan et al., 2012; Figures 1E–G). It is required for healthy sensory hair cell development in a cyclical sequential fashion (Fritzsch et al., 2006; Kageyama et al., 2019; Tateya et al., 2019). *Atoh1* can induce the naïve epithelial cells into hair cells, but the viability of those cells is limited (Jahan et al., 2018; Atkinson et al., 2019). Beyond neonatal cell conversion (Mantela et al., 2005; Kelly et al., 2012; Koehler et al., 2017; McGovern et al., 2019) using pluripotent stem cells (van der Valk et al., 2021; Zine et al., 2021) to make novel cochlear hair cells that are inner- and outer hair cell-specific is beyond our current ability. Cellular deviations are regularly observed in aged materials and almost all aspects of the cell, including nuclear structure, cellular state, mitochondria, genetic material, and protein function that reduce the outer and inner hair cells with time (Kersigo and Fritzsch, 2015; Chessum et al., 2018; Wiwatpanit et al., 2018; Driver and Kelley, 2020; Filova et al., 2020; Herranen et al., 2020). Notably, several later transcription factors interact to maximize the generation of cochlear hair cells (Chen et al., 2021). Approximately 300 genes are identified and associated with ARHL (Bowl et al., 2017). *TMC1/2* is vital for the normal formation of hearing function (Yoshimura et al., 2019; Erives and Fritzsch, 2020), as well as *Cdh23* and *Pcdh15*, which together form the tip links of stereocilia (Elliott et al., 2018; Qiu and Müller, 2018).

Outer hair cells degenerate before inner hair cells in older humans and mice (Spongr et al., 1997; Kane et al., 2012). Specifically, early loss of OHC is pronounced in specific mutations, such as inactivated *Manf* or loss of *Atg7* (Herranen et al., 2020; Perkins et al., 2020; Zhou et al., 2020). Loss of OHCs occurs at different degrees depending on mouse strains. For example, ~80% of OHCs are lost by 26 months of age in C57bl6 mice. In contrast, only ~50% are lost in CBA mice by 26 months. Indeed, in CBA strains, ~100% of hair cells are intact until 18 months. Among the first early loss that affects IHCs, are specific mutants such as *Srrm3/4* (Nakano et al., 2012, 2020) followed by loss of *Cdc42* (Ueyama et al., 2014) and *Arhgef6* (Zhu et al., 2018). Loss of OHC, followed by IHC using a novel diphtheria toxin, was shown in postnatal mice (Tong et al., 2015).

In humans, a loss of OHCs is followed by IHCs that progresses around 65 years of age (Nadol Jr and Xu, 1992; Rauch et al., 2001; Liu et al., 2021). A most detailed description was recently provided that shows a comparable loss of OHCs and IHCs (Wu et al., 2021) that reduces IHCs ~50% in the base of ~75-year-old humans, compared to ~40% in the same region of OHCs (Figures 1C,D). Interestingly enough, in older people with noise-induced hearing loss (NIHL) and/or age-related hearing loss (ARHL), there is a progressive loss of OHC in the base. In contrast, the nearby IHCs remain near normal. Compared to the high-frequency loss, progressive ~50 dB SPL at about 75-year-old to ~60 dB SPL affects nearly 85-year-old or older humans (Figure 1B). Unfortunately, much of the current biological restorative strategies focus on the earliest induction of new hair cells, knowing well that the delayed loss of cochlear hair cells happens about 60 years later (Figure 1A).

Generating vestibular and cochlear hair cells from human pluripotent stem cells have begun the earliest major steps, as mentioned above (Koehler et al., 2017; Lahlou et al., 2018; Patel et al., 2018; Romano et al., 2021; van der Valk et al., 2021; Zine et al., 2021). To date, no one has succeeded in producing both types of the organ of Corti sensory hair cells *in vitro* in the correct proportion and disparity distribution needed for the restoration of hair cells within the organ of Corti. Many issues remain open, but we have no solution at hand, turning the feasibility of producing hair cells with following repopulation of the damaged organ of Corti into a future option (Liu et al., 2021). Restoration of the flat auditory non-sensory epithelium between the lingering patches of hair cells (Pirvola et al., 2002; Kiernan et al., 2005; Soukup et al., 2009; Pan et al., 2012; Duncan and Fritzsch, 2013; Herranen et al., 2020) also requires restoration of innervation to the newly molded hair cells. Concentrating on the proliferation and transdifferentiating of undistinguishable epithelial cells of the flat epithelium requires several steps that need to be taken before precursor cells can respond with genes like *Sox2* and *Atoh1* (Dabdoub et al., 2008; Nyberg et al., 2019; Dvorakova et al., 2020; Yamoah et al., 2020). Appropriate hair cell differentiation in the aging cochlea is the prime objective of hair cell regeneration. Artificial gene governing networks (Ausländer et al., 2012; Sedlmayer et al., 2018; Xie and Fussenegger, 2018; Krawczyk et al., 2020) can be used to drive *Sox2* and *Atoh1* in an oscillatory dynamic manner in BMP4-positive cells. We predict a cargo load of 25 kb is required for this oscillatory gene regulation, well within the choice of the helper-dependent adenovirus (HdAd; 37 kb). It assumes that BMP4 expressing cells can activate and the generation of the LuxR protein will enable the activation of the LuxI, AiiA, and Sox2 proteins. Oscillation will initiate the level of Sox2 gene expression, activating *Atoh1* gene (Neves et al., 2012; Alonso et al., 2018). The expression of the Atoh1 protein can act as positive feedback on its enhancer (Pan et al., 2012) and can provide a negative feedback loop back onto *Sox2* (Dabdoub et al., 2008; Kempfle et al., 2016; Yamoah et al., 2020). Once hair cell differentiation has initiated, *BMP4* expression can be reduced using a negative feedback loop (Lewis R. M. et al., 2018). Eventually, the gene regulation system will shut down after time, leaving feedback loops in the differentiating hair cells to complete the process. Hair cell formation is required to be induced in all age and classes of mice, from young to adult, requiring a scalable expression to take advantage of using synthetic biology to directly repopulate the flat epithelium from undifferentiated supporting cells into cochlear hair cells.

In summary, the progressive loss of cochlear hair cells depends on a large number of genes (Liu et al., 2021) that results in the flat auditory epithelium and requires a re-establishment of normal hair cells for hearing restoration. Alternative approaches, such as incorporating induced hair cells into the cochlea, are challenging. In this case, the hair cells would die once they are exposed to the endolymphatic fluid containing high potassium levels (~140 mM). Early development of hair cells is possible, but the induction of novel hair cells to replace lost hair cells in old mice and humans remains a future biological treatment option.

## Spiral Ganglion Neurons Develop Independent of Hair Cells and Cochlear Nuclei

The sensory neurons of the cochlea, the spiral ganglion neurons (SGNs), depend on *Neurog1* (Ma et al., 1998, 2000; Elliott et al., 2021b) and are myelinated by Schwann cells (Mao et al., 2014; Kersigo et al., 2021). Neurons begin proliferation in a base-to-apex progression around embryonic days E10–12 in mice (Ruben, 1967; Matei et al., 2005), equivalent to gestational week (GW10) in humans (Locher et al., 2013). Central fibers reach the cochlear nuclei at about E12.5, followed by peripheral innervation of hair cells at E14.5 (Yang et al., 2011; Schmidt and Fritzsch, 2019).

**Figure 2:**
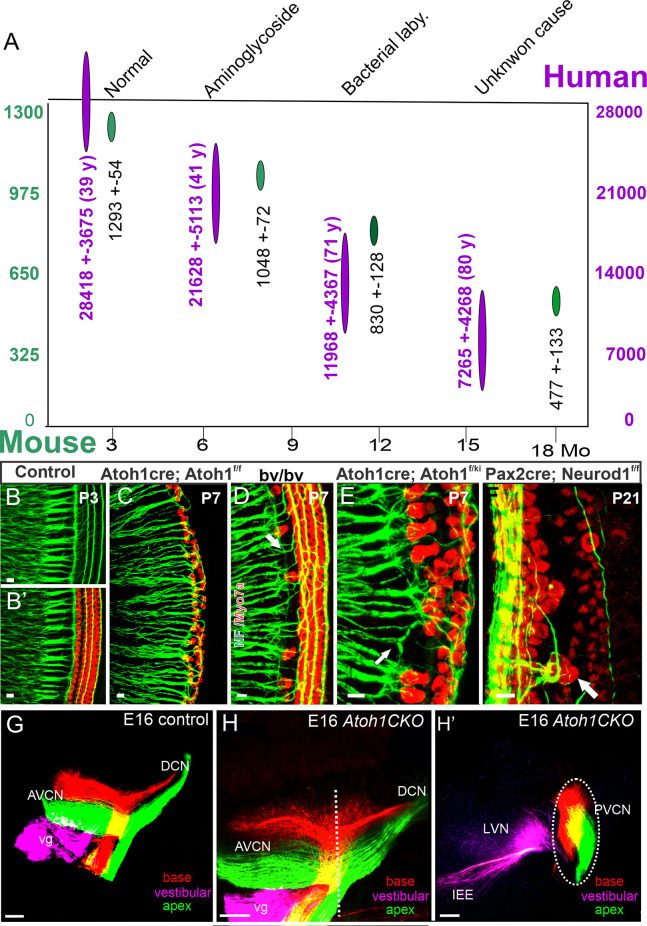
A loss of neurons with time reduces the ~ 28,000 SGNs in ~40-year-old humans to ~25% in 80-year-old humans (**A**, purple). A similar loss is shown for ~1,300 SGNs and reduces to ~40 in 18 month-old mice (**A**, green). Immunoreaction of labeling for neuron markers validates the inner ear afferents in control mice **(B,B’)**. This is compared with *Atoh-cre;Atoh1^f/f^* ‘self-termination mice **(C)**, Bronx waltzer (*bv/bv*) **(D)**, *Atoh-cre;Atoh1^ki/f^* **(E)**, and Pax2-cre;*Neurod1* **(F)**. Central projections reach the anterior (AVCN) and dorsal (DCN) cochlear compared to controls **(G)** and *Atoh*1 C*KO* mice **(H)** that show a tonotopic organization of SGN central projection, despite the absence of cochlear hair cells **(H’)**—modified after Nadol Jr (1990); White et al. (2000); Jahan et al. (2018); and Filova et al. (2020). Scale bars: 10 μm **(B–F)** and 100 μm **(G–H)**.

The genes, *Eya1* (Xu et al., 2021), *Sox2* (Dvorakova et al., 2020), *Shh* (Muthu et al., 2019), *Pax2* (Bouchard et al., 2010), *Lmx1a/b* (Chizhikov et al., 2021), *Dicer/miR-183* (Kersigo et al., 2011), *Fgf7/10* (Pauley et al., 2003; Urness et al., 2018), and *Neurog1* (Ma et al., 1998, 2000; Elliott et al., 2021b), among others, are required to induce the formation of spiral ganglion neurons, as well as cochlear nuclei. Downstream of *Neurog1*, a subset of transcription factors are upregulated to promote SGN differentiation, including *Neurod1* (Jahan et al., 2010a; Macova et al., 2019), *Pou4f1* (Huang et al., 2001), *Gfi1* (Matern et al., 2020), *Isl1* (Radde-Gallwitz et al., 2004; Chumak et al., 2016), and others. Additional factors are also required to maintain spiral ganglion neurons as they project to the cochlear nuclei (Brooks et al., 2020). Expression of the neurotrophins, *Bdnf* and *Ntf3*, are needed for maintaining SGNs (Yang et al., 2011; Fritzsch et al., 2016), and in their absence, no SGNs survive. In the absence of SGNS, the ear initially develops near normally; however, after approximately 2 months, cochlear hair cells degenerate (Kersigo and Fritzsch, 2015). In addition, an early loss of SGNs results in a reduced number of cochlear nuclei neurons (Figures 2B,C) (Dvorakova et al., 2020; Filova et al., 2020), consistent with an early loss in chicken (Levi-Montalcini, 1949). However, unlike in neonates, loss of the ear has less of an effect on cochlear nuclei survival in older mice, suggesting a critical window (Rubel and Fritzsch, 2002; Harris and Rubel, 2006). Our recent data show that vestibular afferents from transplanted mouse ears can innervate the vestibular nuclei of chickens, thus providing a novel approach to eventually replace ears (Elliott and Fritzsch, 2018). Currently, though, cochlear implants are the only means to restore hearing following a sensorineural hearing loss, so long as enough SGNs survive in humans to transmit the sound stimulation from the ear to the cochlear nucleus (Gantz et al., 2005, 2018). In cats, stimulating the ears will reform the cochlear nuclei and develop large endbulbs of Held (Ryugo et al., 2005) and this likely also happens in humans (Kral et al., 2013). Interestingly enough, a set of miR’s show a progressive reduction in cochlear nuclei (Krohs et al., 2021) that acts in parallel to *Dicer* dependent mutations (Kersigo et al., 2011).

The development of SGNs is independent of both cochlear nuclei and cochlear hair cells (Rask-Andersen et al., 2005). Studying the growth and differentiation of SGNs could inform the steps needed to induce otic progenitors to replace lost neurons (Song et al., 2017, 2019; Karlsson et al., 2019). SGN projections follow a simple tonotopic organization (Figures 2G,H,H’): basal projection of the cochlea reaches the most dorsal part of the cochlear nuclei, whereas the apex reaches the ventral aspect of the cochlear nuclei (Muniak et al., 2016; Fritzsch et al., 2019). Following the loss of *Neurod1*, there is a reduction in the number of SGNs and a reorganization of these neurons that result in mixed innervation of auditory and vestibular targets and also a loss of tonotopic organization in the cochlear nuclei (Macova et al., 2019; Filova et al., 2020). A somewhat similar reorganization results from the loss of *Nrp2* that shows a disorganized central cochlear projection (Lu et al., 2014; Schmidt and Fritzsch, 2019). Interestingly, SGNs project to the cochlear nuclei in the absence of *Atoh1* (Fritzsch et al., 2005) where they maintain their tonotopic representation of the periphery (Elliott et al., 2017). In addition, SGNs project peripherally in the absence of *Atoh1* and *Pou4f1* (Xiang et al., 2003) to reach the flat epithelium in the absence of cochlear hair cells (Pauley et al., 2008). The latter demonstrates that SGNs form in the absence of hair cells, suggesting long-term retention of the ’de-innervated’ neurons (Figure 2A) in humans (Nadol Jr, 1990; Nadol Jr and Xu, 1992), cats (Stakhovskaya et al., 2008), and mice (White et al., 2000) whereas neurons of rats degenerate faster within months after the loss of all hair cells (Alam et al., 2007).

Two types of SGNs neurons are known, type I and type II, to innervate the inner hair cells (Petitpré et al., 2018; Shrestha et al., 2018; Sun et al., 2021) and the outer hair cells (Shrestha and Goodrich, 2019; Elliott et al., 2021a), respectively. Innervation of the hair cells occurs in a progression, starting at E18 (Fritzsch et al., 1997) and finishing at about P14 (Petitpré et al., 2018; Shrestha and Goodrich, 2019). In the absence of cochlear hair cells, projecting peripheral neurites of SGNs reach out (Xiang et al., 2003; Fritzsch et al., 2005; Pauley et al., 2008; Pan et al., 2012; Tong et al., 2015), but do not expand beyond the flat auditory epithelium (Shibata et al., 2010). As mentioned above, there is a reduced number of neurons after *Neurod1* deletion and an effect on hair cells in the cochlear apex (Jahan et al., 2010b; Macova et al., 2019). In addition, loss of *Neurod1* results in the conversion of some neurons into hair cells after upregulation of *Atoh1* (Elliott et al., 2021b). Furthermore, in *Neurod1* mutant mice, some OHC are converted into IHC-like cells (Figure 2F) and are innervated by putative type I SGNs (Jahan et al., 2018; Filova et al., 2020). Reorganization of innervation has been shown in *Srrm4* mutant mice. In these mice, IHCs are primarily absent (Figure 2D), and type I SGNs innervate the OHCs instead can enforce some of the type I to innervate IHC instead of OHC (Figures 2B,C) (Nakano et al., 2012; Jahan et al., 2018). Replacement of one allele of *Atoh1* with *Neurog1*, in which the other *Atoh1* allele is conditionally knocked out, can lead to the formation of near-normal hair cells (Figure 2E) that also receive innervation (Jahan et al., 2015). A unique defect of SGNs is observed at a certain reduction level of specific stimulation in mice (Kujawa and Liberman, 2009) that requires additional information in humans (Wu et al., 2021).

In summary, SGNs project centrally to the cochlear nucleus and peripherally to the hair cells. SGNs show a tonotopic organization to the cochlear nuclei even in the absence of hair cells, which is beneficial for cochlear implants. Likewise, in the absence of hair cells, long-term retention of SGNs is demonstrated and ramified within the “flat auditory epithelium” that could possibly be re-connected with novel hair cells in the near future.

## Cochlear Nuclei Depend on Transcription Factors That May Change with Age

**Figure 3:**
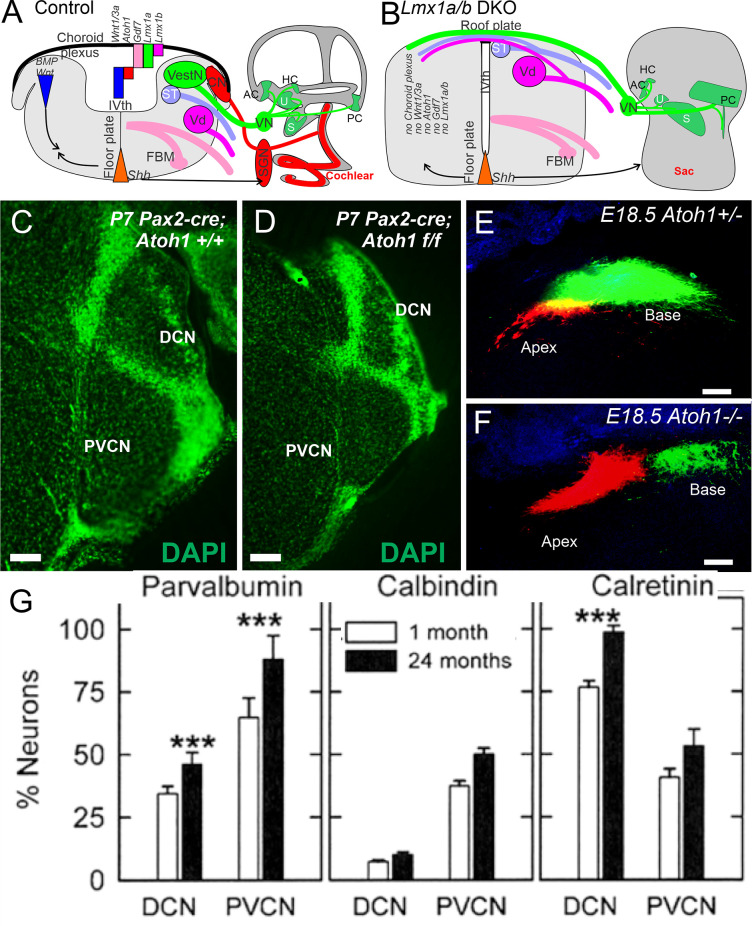
Cochlear nuclei depend on *Lmx1a/b*, upstream of *Gdf7*, *Atoh1*, and *Wnt3a* **(A)**. In the absence of *Lmx1a/b* neither cochlear hair cells, spiral ganglion neurons, nor cochlear nuclei develop **(B)**. The choroid plexus is replaced by a roof plate that allows fibers to cross to the contralateral side **(A,B)**. Cochlear nuclei are smaller in a conditional deletion of *Atoh1* using Pax2-cre** (C,D)**. In *Atoh1*-null mice, there is a normal central projection of SGNs without either peripheral hair cells or cochlear nuclei **(E,F)**. Expression of Parvalbumin, Calbindin and Calretinin showing upregulation in the cochlear nuclei at the level of DCN and PVCN areas that increase in some but not all neurons **(G)**. Modified after Idrizbegovic et al. (2006); Elliott et al. (2017); Elliott et al. (2021b); and Chizhikov et al. (2021). Scale bars: 100 μm **(C–F)**. *** indicates significance.

The cochlear nucleus is the first information processing center for auditory information within the central nervous system and provides output to the superior olivary cells (SOC) and the inferior colliculi (IC) (Malmierca and Ryugo, 2011; Oertel and Cao, 2020). It comprises three subnuclei: anteroventral cochlear nucleus (AVCN) from rhombomere 2 and 3 (r2, 3), posteroventral cochlear nucleus (PVCN; r4), and dorsal cochlear nucleus (DCN, r5). Most cochlear nuclei neurons exit the cell cycle around E10–14 except for the delayed formation of granule cells that finish at E18 in mice (Pierce, 1967). *Atoh1* is a major bHLH gene that defines the cochlear nuclei. It is expressed in regions of the rhombic lip of the hindbrain that contribute cells to the cochlear nucleus. Conditional deletion of *Atoh1* in r3/5 impaired cochlear nucleus formation in those regions (Maricich et al., 2009)., without *Atoh1*, there is no formation of cochlear nuclei (Wang et al., 2005; Fritzsch et al., 2006; Ray and Dymecki, 2009). However, as mentioned earlier, in the absence of *Atoh1*, a near-normal central projection develops (Figures 3E,F) of SGNs (Elliott et al., 2017). Notably, a delayed loss of *Atoh1* can be driven by *Pax2* (Figures 3C,D). Upstream of *Atoh1* is the expression of *Lmx1a/b* (Chizhikov et al., 2021). Without expression of *Lmx1a/b*, there is a loss of *Gdf7* (Lee et al., 2000), *Atoh1*, and *Wnt3a* expression, and there is no formation of the choroid plexus (Chizhikov et al., 2021; Elliott et al., 2021b). In addition, without *Lmx1a/b*, all aspects of the cochlea are eliminated beyond an undifferentiated sac, including no differentiation of cochlear hair cells or SGNs (Figures 3A,B). Additional transcription factors have been identified for cochlear nuclei formation (Milinkeviciute and Cramer, 2020). For instance, *Hoxb1* is expressed in r4 in the PVCN and parts of the DCN (Di Bonito et al., 2017).

The cochlear nuclei have defined numerous cell types based on morphological and physiological criteria (Oertel and Cao, 2020). Synapses from type I SGNs onto bushy cells in the AVCN have structural adaptations that receive the endbulbs of Held; the endbulb of Held permits highly secure signaling between the auditory periphery of SGN and bushy cells (Manis et al., 2012; Caspary and Llano, 2018; Syka, 2020; Wang et al., 2021). Losing proper auditory innervation that leads to a simplification of the endbulb termination (Muniak et al., 2016). Local inhibitory cells in the cochlear nucleus are known as either glycinergic (e.g., D-multipolar, vertical, and cartwheel) or as GABAergic [stellate and Golgi; (Young and Oertel, 1998; Oertel and Cao, 2020)] that receive the SGN projections. Genomically are clustered as miR-183 and miR-96 that reduces the volumes of auditory hindbrain nuclei. Electrophysiological analysis shows that the calyx of Held synapses on the medial nucleus of the trapezoid body (MNTB) demonstrated strongly altered synaptic transmission in young-adult mice (Krohs et al., 2021).

Aging-related changes are known to a certain extent, followed in neuronal morphology and biochemistry in the cochlear nucleus and SOC. The size of the AVCN remains constant with aging, whereas its cell density decreases with age. Moreover, cellular density correlates with aging-related loss of cochlear sensory neuron input. In addition, the cellular density diminishes by about 15% at 7 months of age in certain mice (C57BL/6). Still, it does not show a reduction of cellular density until the 2nd year of life in CBA/CaJ mice (Willott et al., 1987). The reduction of cellular density is complemented by a drop in the size of synaptic terminals by AVCN neurons (Briner and Willott, 1989; Helfert et al., 2003). The volume of the DCN and cell number and size of aging-related decreases have been detected in C57BL/J, suggesting that the losses are related to hearing loss. Changes have been detected in the octopus cell area of the PVCN. Interestingly enough, changes seen in both C57BL/J and CBA/CaJ mice indicate a different aging-related effect (Willott and Bross, 1990; Jalenques et al., 1995). The morphology of the cochlear nucleus in humans with presbycusis suggests an increase in the total cell number and number of multipolar and granule neurons in the cochlear nucleus with aging (Hinojosa and Nelson, 2011), a contradictory result that requires additional work (Syka, 2020).

The calcium-binding proteins parvalbumin, calbindin, and calretinin play a leading role in buffering intracellular calcium during cellular stress in all cochlear nuclei (Lohmann and Friauf, 1996). For example, calretinin-, calbindin-, and parvalbumin-positive cells are perceived in the DCN and PVCN of aged mice; this increase of the three proteins is correlated with hair cell loss (Idrizbegovic et al., 2003; Caspary and Llano, 2018). Recent data suggest the total number of neurons in the DCN and PVCN is decreasing while the proportion of parvalbumin- and calretinin-positive neurons (Figure 3G) is increasing (Idrizbegovic et al., 2006; Syka, 2020). Hearing loss goes beyond the three proteins that are the only factor in these increases. Moreover, induced hearing loss may cause a *lessened* effect of calretinin-positive neurons in layer III of the aged DCN, innervating the fusiform neurons (Zettel et al., 2003; Syka, 2020) in the human cochlear nucleus. Note that all three calcium-binding proteins decrease progressively from adult to old age (Sharma et al., 2014). The differences in intracellular calcium mobilization may play a role to confirm the differences between the human and mouse data (Ibrahim and Llano, 2019).

Changes with aging in the cochlear nucleus are known to degrade the spiking activity in opposition to temporally patterned impetuses. The Fischer 344 rat showed aging-related decreases in glycine binding sites. In addition, receptor subunits in the cochlear nucleus are declining (Wang et al., 2009; Caspary and Llano, 2018). Reduction and loss of inhibition are most likely mirrored in cochlear nucleus neurons’ firing possessions. Thus, cartwheel and fusiform cells show increased acoustically driven responses in the aging cochlear nucleus. This activity will reflect the loss of inhibition (Caspary et al., 2006; Caspary and Llano, 2018). Physiologically, numerous studies show the temporal fidelity that breaks down at the synapse between cochlear nerve fibers and bushy cells in old mice (Xie, 2016; Xie and Manis, 2017; Wang et al., 2021). These data suggest multiple instruments may be responsible for the aging-related temporal spreading of cochlear nucleus neuronal responses from temporally patterned sounds. Moreover, the first longitudinal investigation of different type Ia is positive for calretinin and innervates the bushy neurons.

In summary, we show that cochlear nuclei depend on *Lmx1a/b* and *Atoh1* and require further studies with heterozygotes to identify the long-term defects in mice. Preliminary data suggest a distinct effect of upregulation of parvalbumin and calretinin that could upregulate the relative calbindin expression and suggest a long-term reduction of calretinin that innervates the bushy cells. No new cochlear nuclei are needed as they survive in the absence of SGNs.

## Superior Olive Complex Is Affected by Various Auditory System Losses

The superior olivary complex (SOC) is the second major station in the ascending auditory pathway, which consists of the first input at which information from the two ears is combined for binaural information in the central auditory system. The SOC nuclei involved in processing ascending information are the medial superior olive (MSO), lateral superior olive (LSO), and medial nucleus of the trapezoid body (MNTB; Marrs et al., 2013). These three major SOC nuclei play a critical role in sound localization ability, which is known to diminish with age (Caspary and Llano, 2018; Syka, 2020). In mice, neurons are born between embryonic days E9–14 with the latest differentiated neurons in the LSO (Pierce, 1973).

Recent data suggest that the SOC complex is generated in part by dorsal neurons from *Atoh1*-positive cells that identify their contribution to distinct migrating SOC cells (Maricich et al., 2009; Marrs et al., 2013; Di Bonito et al., 2017; Lipovsek and Wingate, 2018). In addition to *Atoh1*, there is a combined expression of *Wnt3a* and *En1* (Jalabi et al., 2013; Altieri et al., 2015; Chizhikov et al., 2021) that are important for the SOC. A noticeable difference is in the SOC of the chicken that generates more caudal nuclei (Lipovsek and Wingate, 2018). A clear progression in Calbindin, Calretinin, and Parvalbumin expression that require a generation of SOCs is demonstrated by tracing and immunological data (Lohmann and Friauf, 1996; Kandler et al., 2020).

Very few studies have investigated variations in the MSO, LSO, and MNTB, particularly with aging. For example, Godfrey et al. found that there were no changes in the dry weight of the superior olive complex, but that was significantly reduced in glutamate, glycine, and GABA (Godfrey et al., 2017). Moreover, there is a reduction of about 25% in the level of neurotransmitters with aging in the auditory system. The size of glycinergic and GABAergic cell size has become smaller in the LSO of gerbils of aging (Gleich et al., 2004). Furthermore, decreases in synaptic terminals have been reduced in MNTB in Sprague-Dawley rat with age (Casey and Feldman, 1982, 1985, 1988). An increase in parvalbumin staining in the MSO, but not LSO or MNTB, is reported in primates with aging (Gray et al., 2013). Changes seem to correlate with deviations in peripheral hearing, implying that parvalbumin upregulation may be a part of the mechanism to compensate for the loss of peripheral input from neurons. Beyond significant changes in the synaptic organization in superior olives that have provided few aging-related physiological changes *in vivo* compared with changes in the ABR threshold (Ibrahim and Llano, 2019; Syka, 2020). Older miR-183/96 require studying in much older mice to study the long-term defects (Krohs et al., 2021).

**Figure 4:**
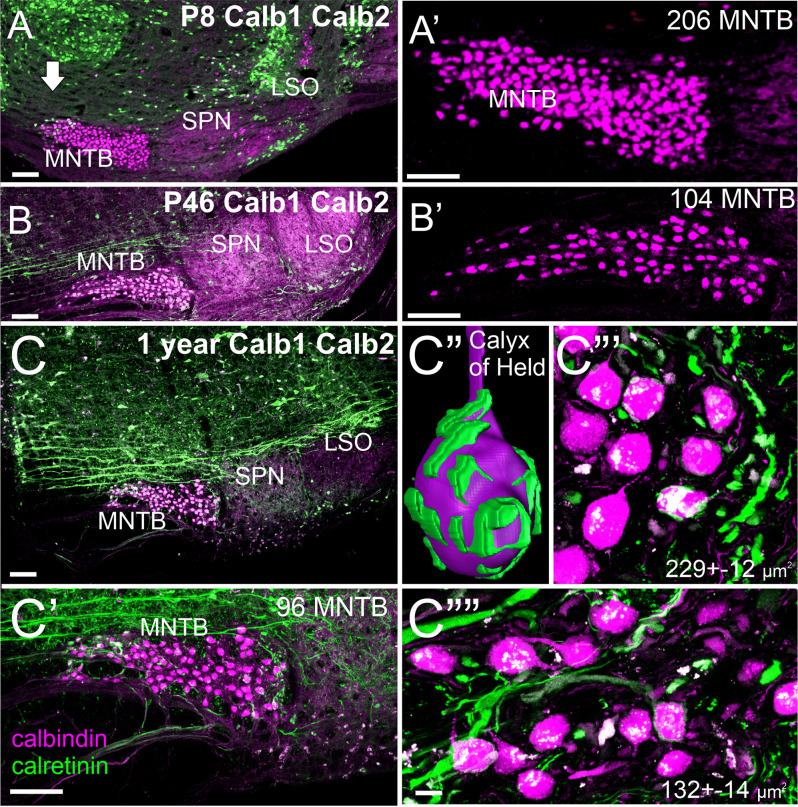
Early expression of *Calbindin* (*Calb1*) and *Calretinin* (*Calb2*) expression in the MNTB at P8 **(A,A’)**. *Calb1* expression increases in the LSO by P46 **(B,B’)**. At 1 year of age, there is minimal *Calb1* expression in the LSO and SPN. *Calb2*-positive fibers form a calyx of Held in the MNTB **(C–C””)**. Within the MNTB, there are larger *Calb1*-positive cells close to the cochlear nucleus (~229 μm^2^) compared to much smaller cells closer to the midline (~132 μm^2^). Our expression shows upregulation of *Calb2* in the MNTB (compare P8 with P46) that progressively upregulates with *Calb1*. Note that fewer MNTB are shown in a single section that likely comes about through lateral distributions. P: postnatal, Bar indicates 100 μm in **(A–C,C’)**, 10 μm in **(C”’,C””)**.

Our initial set of calretinin and calbindin data suggests a slight reduction in the number of MNTB cells (Figure 4) consistent with data in rats (Casey and Feldman, 1988). Using a gene regulation of two distinct nuclei showing a fast up- and down-regulation for three nuclei that are all positive for calbindin (Figures 4A–C). Whereas the MNTB is calbindin-positive early and keeps this expression, the SPN and LSO show a strong early up-regulation of calbindin followed by near regular expression of calbindin at 1 year of age. An interesting size of MNTB differs between 1 year-old mice: more significant more lateral neurons are becoming more smaller in the medial MNTB (Figures 4C,C””). Individual neurons can be traced by calretinin to form a calyx of Held (Figure 4C”) that resembles the endbulb of Held of bushy cells (Wang et al., 2021). While all calyx receives innervation from the contralateral cochlea, the size of innervation suggests that smaller MNTB neurons have fewer calyces (Figures 4C”’,C””). Further work is needed to detail the loss in mice using calretinin, calbindin, and parvalbumin to investigate the effects in mice 24 months or older.

In summary, we know that the migrated SOC nuclei are derived from the dorsal rhombomere. Our preliminary set of calretinin and calbindin data suggest that different complexity and size of MNTB neurons correlate with the size of neurons with aging.

## Conclusions

Our overview shows the earliest loss of cochlear hair cells, spiral ganglion neurons, cochlear nuclei, and associated superior olivary complex: Nearly all four auditory systems depend on *Lmx1a/b, Atoh1*, and *Neurog1* expression. Later, a progressive loss of hair cells occurs with aging, followed by a spiral ganglion neuron reduction to ~40% in humans and mice. In contrast, early losses of cochlear nuclei have been well described, showing changes in different proteins for survival. How much of the changes are related to compensation for peripheral hearing loss remains an open question, and the impact of adaptation on sound perception remains a subject for future investigations. Auditory brainstem neurons may be less vulnerable to oxidative stress, which may have high metabolic demands based on neurons’ high firing rates. Thus, different mechanisms may interpret for aging declines, in particular, inhibition that affects the auditory pathway. We suggest that the loss of inhibition may, in part, be responsible for temporal fidelity or may be affected by sound localization or may produce dysregulated plasticity. Future work will be of help to explain the causes of changes of aging that ultimately affect the central auditory system that will lead to novel therapeutic objectives to perfect such changes by adding new hair cells and connecting them with remaining SGNs to connect the cochlear nuclei. How much cochlear nuclei and SOC and IC neurons can plastically revert in sizes and complexity of innervation remains to be seen. Additional investigations to uncover the complex reasons for changes in the central auditory system will contribute to developing new healing targets to alleviate sensory ARHL and associated cognitive decline.

## Author Contributions

This work was conceived by AZ and BF, final revision was drafted by KE, ENY and BF, reviewed by AZ and ENY. All authors edited the final draft in collaboration and approved the submitted version. All authors contributed to the article and approved the submitted version.

## Conflict of Interest

The authors declare that the research was conducted in the absence of any commercial or financial relationships that could be construed as a potential conflict of interest.

## Publisher’s Note

All claims expressed in this article are solely those of the authors and do not necessarily represent those of their affiliated organizations, or those of the publisher, the editors and the reviewers. Any product that may be evaluated in this article, or claim that may be made by its manufacturer, is not guaranteed or endorsed by the publisher.
